# A lifestyle pattern during adolescence is associated with cardiovascular risk markers in young adults: results from the DONALD cohort study

**DOI:** 10.1017/jns.2021.84

**Published:** 2021-10-12

**Authors:** Maike Elena Schnermann, Christina-Alexandra Schulz, Christian Herder, Ute Alexy, Ute Nöthlings

**Affiliations:** 1 Institute of Nutrition and Food Sciences, Nutritional Epidemiology, University of Bonn, Bonn, Germany; 2 Institute for Clinical Diabetology, German Diabetes Center, Leibniz Center for Diabetes Research at Heinrich Heine University Düsseldorf, Düsseldorf, Germany; 3 German Center for Diabetes Research, Düsseldorf, Germany; 4 Division of Endocrinology and Diabetology, Medical Faculty, Heinrich Heine University Düsseldorf, Düsseldorf, Germany

**Keywords:** Adolescent lifestyle, Cardiovascular disease risk, Combined lifestyle factors, Lifestyle pattern, Young adults

## Abstract

Lifestyle score approaches combining individual lifestyle factors, e.g. favourable diet, physical activity or normal body weight, showed inverse associations with cardiovascular disease (CVD) risk. However, research mainly focussed on adult behaviour and is scarce for vulnerable time windows for adult health like adolescence. We investigated associations between an adolescent lifestyle score and CVD risk markers in young adulthood. Overall, we analysed 270 participants of the open DONALD cohort study with 1–6 complete measurements of five lifestyle factors (healthy diet, moderate-to-vigorous physical activity, sedentary behaviour, sleep duration and BMI standard deviation score) during adolescence (females: 8⋅5–15⋅5 years and males: 9⋅5–16⋅5 years). Multivariable linear regression models were used to investigate the prospective association between the adolescent lifestyle score (0–5 points) and CVD risk markers in young adulthood (18–30 years). On average, participants obtained a mean adolescent lifestyle score of 2⋅9 (0–5) points. Inverse associations between the adolescent lifestyle score and waist circumference, waist-to-height ratio and percentage of body fat were observed (4⋅1, 4⋅1 and 9⋅2 % decrease per 1 point increase in adolescent lifestyle score, respectively, *P* < 0⋅05). For the remaining CVD risk markers (glucose, blood lipids, blood pressure and a proinflammatory score), no associations were observed. A healthy adolescent lifestyle is particularly associated with CVD risk-related favourable anthropometric markers in adulthood. A more comprehensive understanding of lifestyle patterns in the life course might enable earlier, targeted preventive measures to assist vulnerable groups in prevention of chronic diseases.

## Introduction

Associations between lifestyle factors and cardiovascular disease (CVD) risk are well known in adults. Studies investigating lifestyle scores, i.e. combining individual lifestyle factors such as adherence to a specific diet, physical activity, smoking behaviour, alcohol consumption, body mass index (BMI)^(1–4)^, sleep quality, social activities^(5,6)^ and screen time^(7)^ contributed in a promising way to the current evidence. As such, single factors are part of an overall lifestyle, which is often described as the sum of health-related factors^(8,9)^. Using a multidimensional lifestyle pattern approach, various risk factors are simultaneously considered as well as the interaction between them^(10–12)^. When considering such multidimensional patterns, cut-off values of single score factors can be either based on established references^(13,14)^ or on population-specific cut-offs^(15)^ and summed up to an overall score. In a recent meta-analysis of twenty-two cohort studies, adherence to a healthy lifestyle was associated with a 66 % reduction in total CVD risk^(12)^.

Also for adolescence, current recommendations for different lifestyle behaviours exist. Individuals are encouraged to eat a healthy diet^(16–18)^, have at least 60 min of moderate-to-vigorous activity per day^(19)^, less than 120 min of daily sedentary time^(20)^, a sleep duration according age-specific recommended hours^(21)^ or a healthy body composition^(22)^. Adolescent lifestyle behaviours are important for health and disease in adulthood, as behaviours established in adolescence might track into adulthood^(23)^. Furthermore, it has been shown that a healthy diet and a predominantly active lifestyle during adolescence have an impact on various health outcomes in adulthood. Healthy dietary patterns were associated with CVD risk markers such as blood pressure^(24,25)^, blood lipids^(25,26)^ or clustered cardiometabolic risk^(27)^. In addition, several studies investigated the effect of physical activity on cardiometabolic risk. Inverse associations between a predominantly active lifestyle and body composition^(28,29)^, and triglycerides have been found^(30,31)^. The results regarding sedentary behaviour (i.e. television watching or video gaming) are more inconclusive, as some studies reported inverse associations between a sedentary lifestyle and cardiometabolic risk^(32,33)^, whereas others reported no associations^(30,34)^. Moreover, short sleep duration was shown to be negatively associated with CVD risk markers in a systematic review^(35)^, which was recently supported by a meta-analysis^(36)^. An unfavourable body composition during adolescence has been shown to be positively associated with later risk of coronary heart disease^(37)^ or CVD^(38)^. Consequently, an unhealthy diet, inactivity, inadequate sleep duration or an unfavourable body composition earlier in life may be maintained later in life, leading to an increased risk of chronic diseases ^(24,26–33,35–38)^.

Up to now, only few studies analysed the relationship between adolescent lifestyle patterns and cardiovascular outcomes in adulthood^(39,40)^. Thus, it seems relevant to develop a lifestyle score for adolescents and to investigate its relationship with health-related endpoints in later life.

Therefore, the aim of the present study was (1) to construct a hypothesis-based lifestyle score for adolescents including diet, physical activity, sedentary behaviour, sleep duration and body composition, and (2) to investigate the relationship between the adolescent lifestyle score and CVD risk markers in early adulthood.

## Research design and methods

### Study design

The DOrtmund Nutritional and Anthropometric Longitudinally Designed (DONALD) Study is an ongoing open cohort study going back to 1985. A detailed study description has been published elsewhere^(41)^. Repeated annual assessments and examinations in childhood and adolescence are conducted and continued in young adulthood. Data on dietary intake, anthropometry and physical activity are collected annually. For adult participants (≥18 years), a fasting blood sampling is taken in addition to the routine assessment with medical examination every 5 years. This study was conducted according to the guidelines laid down in the Declaration of Helsinki and all procedures involving human subjects were approved by the Ethics Committee of the University of Bonn (ethics numbers: 098/06 and 185/20). Written informed consent was obtained from study participants or parents.

### Study population

For the present analysis, participants with at least one measurement of all considered lifestyle factors during adolescence (females: 8⋅5–15⋅5 years and males: 9⋅5–16⋅5 years) and anthropometric and blood measurements of CVD risk markers during adulthood (18–30 years) were selected (*n* 290). Participants were not included if they were pre-term or post-term (<36, or >42 gestation week, respectively, *n* 8), part of multiples (*n* 7) or had a low birth weight (<2500 g, *n* 3), thus sample size was reduced to *n* 272. In addition, participants with missing confounding variables were excluded (*n* 2), resulting in a sample size of 270 participants.

### Assessment of lifestyle variables

Three-day weighed dietary records (3dWR) were used to collected dietary intake. Participants were asked to weigh all foods, dishes and beverages consumed over a 3-d period. If weighing was not possible, data were recorded using household measures. Using the in-house database LEBTAB^(41,42)^, food group intake was calculated. For each 3dWR, a mean value for consumed foods and beverages in g/d was calculated. When dividing g/d by age-dependent serving sizes (Supplementary Table S1) in grams/serving, the number of serving/d results. Data from 3dWR were checked for possible underreporting. Participants were classified as ‘potential underreporters’ if they reported energy intakes at more than half of the available 3dWR that were inconsistent with basal metabolic rates calculated on the basis of Schofield's sex- and age-specific equations^(43)^ and Goldberg's energy intake thresholds^(44)^.

Moderate-to-vigorous physical activity (MVPA) was assessed since 2004 via the interviewer-based, validated Adolescent Physical Activity Recall Questionnaires, which included questions on organised and unorganised activities with their duration and frequency^(45)^. Sedentary behaviour was assessed via a questionnaire, which was used in the nationwide Children and Youth Survey of the Robert Koch Institute and included questions on duration and frequency of sedentary activities during weekdays and on the weekend^(46–48)^. If the value for the weekend was missing, the value of the weekday was used and vice versa. Daily sleep duration was recorded via a questionnaire. To calculate BMI (kg/m^2^), trained nurses measured weight (kg) and height (m) annually without shoes and wearing underwear only. BMI was calculated as the weight (kg) divided by the square of height (m^2^). BMI SDS (standard deviation score) was calculated using the national age- and sex-specific BMI percentiles^(22)^.

### Development of the adolescent lifestyle score in the DONALD study

We developed an adolescent lifestyle score based on five lifestyle factors, i.e. diet, calculated as individual means of the 3dWR, duration of MVPA, sedentary behaviour and sleep, and BMI SDS. ‘Healthy diet’ was based on six food groups, namely fruits, vegetables, whole grains, sugar-sweetened beverages, fish and red meat. A score of 0 or 1 was assigned for each lifestyle factor, whereby 1 point was given when the recommendation was completely fulfilled and 0 when not (Table 1). Additional information can be found in Supplementary material S1.

**Table 1.** Lifestyle factors and scoring system of the lifestyle score

Lifestyle factor and scoring criteria	Points	Recommendation
Overall score	0–5	
Healthy diet		DGE^(16)^, USDA^(17)^, WHO^(18)^
0–2 food groups/d	0	
3–6 food groups/d	1	
MVPA		WHO^(19)^
<60 min/d	0	
≥60 min/d	1	
Sedentary behaviour		Graf *et al.*^(^^20^^)^
8⋅5–11 years: >60 min/d		
12–16⋅5 years: >120 min/d		
8⋅5–11 years: ≤60 min/d	0	
12–16⋅5 years: ≤120 min/d	1	
Sleep duration		AASM^(21)^
8⋅5–12 years: <9 and >11 h/d		
13–16⋅5 years: <8 and >10 h/d		
8⋅5–12 years: 9–11 h/d	0	
13–16⋅5 years: 8–10 h/d	1	
BMI SDS		Kromeyer-Hauschild *et al.*^(22)^
Overweight or underweight	0	
Normal weight	1	

DGE, German Nutrition Society; USDA, United States Department of Agriculture; WHO, World Health Organisation; MVPA, moderate-to-vigorous physical activity; AASM, American Academy of Sleep Medicine; BMI, body mass index; SDS, standard deviation score.

Individual factor scores were then summed up to an overall score ranging from 0 to 5 points, with higher scores indicating a healthier lifestyle. For the lifestyle score during adolescence, the mean value of yearly lifestyle scores was calculated.

To investigate plausibility of the score, we calculated age- and sex-adjusted trends of single lifestyle factors across tertiles of the lifestyle score. As expected, the higher the adolescent lifestyle score, the more fruit, vegetables and whole grains as well as less sugar-sweetened beverages and red meat were consumed (*P*_trend_ <0⋅0001). In addition, a higher adolescent lifestyle score was associated with a longer duration of MVPA and sleep, less time spent sitting and a healthier BMI SDS (*P*_trend_ <0⋅0001). More in-depth information can be found in Supplementary material S2 and Table S2.

### Assessment of CVD risk markers

CVD risk markers included (1) anthropometric markers (waist circumference (WC), waist-to-height ratio (WHtR), percentage of body fat (%BF), systolic blood pressure, diastolic blood pressure), (2) metabolic markers (glucose, cholesterol, HDL-C, LDL-C, triglycerides) and (3) a proinflammatory score combining different biomarkers of inflammation (C-reactive protein (CRP), interleukin (IL)-6, IL-18, adiponectin and leptin). These CVD risk markers were assessed as described before^(49–55)^. Parameters such as height and weight, WC and skinfold thickness as well as blood pressure were measured as described previously^(41,56)^. WHtR (WC divided by height) as well as %BF from four skinfolds according to Durnin and Womersley were calculated^(57)^. As a combination of multiple inflammatory markers might be more predictive than considering individual markers of inflammation^(58)^, we decided to analyse a proinflammatory score as described previously^(50,51)^. Therefore, we standardised all inflammatory parameters by sex (mean = 0, sd = 1), multiplied the anti-inflammatory marker adiponectin with −1 to have the proinflammatory effect, and averaged all.

### Assessment of additional variables

Gestational and birth parameters such as pregnancy duration (weeks), birth size (cm), birth weight (g) and mother's weight gain during pregnancy (kg) were collected at the child's admission to the study via a German standardised pregnancy document (“Mutterpass”). Breastfeeding duration (weeks) was recorded via repeated parental interviews during the first year of life. Additionally, parents were interviewed on socio-economic characteristics such as education, employment and smoking status at regular intervals. Maternal overweight (BMI ≥ 25 kg/m^2^) and participants’ BMI in adulthood were measured every 4 years with the same procedure as for the participants in adolescence. Age at outcome assessment and time between measurement of the score and the risk variables were also taken into account.

### Statistical analysis

Participants were classified into tertiles according to their adolescent lifestyle score to provide a descriptive characterisation of the study participants. Continuous variables were presented as median ± IQR and categorical variables as relative frequencies (%), if not stated otherwise. Trends across the tertiles of the adolescent lifestyle score were tested using age- and sex-adjusted linear regression models.

Not normally distributed outcome variables were log-transformed. For the interpretation of the results, log-transformed variables were back transformed [(exp(ß-estimate) − 1) * 100] to show the percentage change of the dependent variable. A log transformation of the proinflammatory score is not possible due to its values in the negative range. Therefore, we have divided the proinflammatory score into tertiles, with participants in the lowest tertile having the lowest proinflammatory status. Outliers that significantly disturb the normal distribution of the residuals were replaced by the sex-specific value that most closely resembles the distribution. This affected 1 % of the inflammatory biomarkers.

We conducted several analyses to examine the associations between the adolescent lifestyle score and CVD risk markers using multivariable linear regression models. We did not stratify our analysis by sex, because no significant interaction of the adolescent lifestyle score and sex was observed for any CVD risk marker (*P* > 0⋅05). First, we used the adolescent lifestyle score as a continuous variable. The basic model was adjusted for sex and age at outcome assessment. The multivariate adjusted model included potential influencing covariates, which considerably modified the predictor-outcome associations (change in ß-estimates ≥10 %)^(59)^. These were smoking status in the household (never/former/current), exclusive breastfeeding for ≥4 month (yes/no), birth size (cm), maternal overweight (kg/m^2^) and time between measurement of the score and the risk variables (years). In addition, we calculated a conditional model including additionally participants’ BMI in adulthood to examine whether the observed associations may partly be explainable by adulthood body composition. Second, we calculated five modified lifestyle scores based on only four lifestyle factors instead of five, omitting one factor at a time. In this analysis, we adjusted the multivariate adjusted model additionally for the omitted lifestyle factor. To limit the possibility of false positive results, we adjusted *P*-values for multiple testing by controlling the false discovery rate at 5 % according to the Benjamini–Hochberg approach^(60)^. Depending on data availability, we analysed 263 participants with anthropometric risk markers, 228 participants with metabolic risk markers and 154 participants with a proinflammatory score. Power calculations based on partial correlation coefficients indicated that the sample size was large enough to detect associations between exposure and anthropometric risk markers with a power of 98 %. For metabolic risk markers and the proinflammatory score, statistical power was between 18⋅3 and 58⋅4 %.

Additional sensitivity analyses in subsamples of participants who provided (1) at least two measurements of each lifestyle factor (*n* 250) and (2) participants who were not considered as potential underreporters (*n* 244) were performed. All statistical analyses were performed using SAS (Version 9.4; Cary, NC, USA). Statistical significance was defined as a *P*-value < 0⋅05.

## Results

Descriptive characteristics of all 270 participants are shown in Table 2. Slightly more than half of the participants were male (52 %). The overall mean adolescent lifestyle score of all study participants was 2⋅9 (0–5) points. Participants with a higher adolescent lifestyle score were more likely to have higher consumption of fruits, vegetables as well as wholegrain, lower consumption of sugar-sweetened beverages and red meat, were more active and had a normal body weight. Additional information on participants’ reference fulfilment can be found in Supplementary Table S3.

**Table 2.** Basic characteristics^a^ of the 270 study participants across tertiles of the adolescent lifestyle score

	T1 (2⋅0 [0–2⋅6])	T2 (2⋅9 [2⋅6–3⋅2])	T3 (3⋅7 [3⋅3–5])	*P*_trend_^b^
Age at blood withdrawal (years)^c^	19⋅8 (18⋅0–29⋅8)	19⋅7 (18⋅0–28⋅0)	19⋅6 (18⋅0–28⋅7)	0⋅71
Age difference exposure – outcome	5⋅8 (2⋅0)	6⋅0 (3⋅5)	6⋅0 (2⋅9)	0⋅67
Male participants (%)	53⋅3	50⋅0	43⋅3	0⋅68
Baseline parameters				
Birth size (cm)	52 (3)	52 (3)	52 (3)	0⋅62
Parental education (>12 years, %)	65⋅2	77⋅3	75⋅6	0⋅13
Smoking household (yes, %)	21⋅7	17⋅1	12⋅2	0⋅10
Exclusive breastfeeding (>4 month, %)	69⋅6	69⋅3	74⋅4	0⋅49
Maternal overweight (yes, %)	46⋅7	39⋅8	27⋅8	**0**⋅**0090**
Potential underreporter (%)	18⋅5	6⋅8	3⋅3	**0**⋅**0003**
≥2 lifestyle assessments (%)	92⋅4	89⋅8	95⋅5	0⋅48
Dietary intake and lifestyle during adolescence				
Fruits (g/d)	236⋅4 (191⋅5)	268⋅4 (189⋅3)	356⋅9 (276⋅3)	**<0**⋅**0001**
Vegetables (g/d)	106⋅8 (88⋅0)	107⋅1 (74⋅2)	127⋅1 (80⋅9)	**0**⋅**0027**
Whole grain (g/d)	27⋅0 (35⋅9)	29⋅3 (40⋅8)	48⋅6 (50⋅9)	**<0**⋅**0001**
Sugar-sweetened beverages (g/d)	228⋅8 (368⋅1)	164⋅2 (289⋅9)	102⋅4 (165⋅7)	**0**⋅**0007**
Fish (g/d)	10⋅6 (25⋅8)	13⋅8 (20⋅3)	13⋅0 (23⋅0)	0⋅89
Red meat and sausages (g/d)	79⋅3 (57⋅0)	73⋅2 (44⋅4)	67⋅9 (43⋅2)	**0**⋅**0004**
MVPA (min/d)	50⋅3 (40⋅4)	67⋅4 (27⋅8)	75⋅4 (34⋅8)	**<0**⋅**0001**
Sedentary behaviour (min/d)	182⋅4 (60⋅7)	160⋅6 (64⋅3)	124⋅3 (51⋅4)	**<0**⋅**0001**
Sleep duration (h/d)	8⋅5 (1⋅0)	9⋅0 (0⋅8)	8⋅9 (0⋅6)	**0**⋅**0003**
BMI SDS	0⋅7 (1⋅9)	0 (1⋅1)	−0⋅1 (1⋅0)	**<0**⋅**0001**

MVPA, moderate-to-vigorous physical activity; BMI, body mass index; SDS, standard deviation score. Values displayed in bold are significant at 0.05 significance level.

a Data shown as median (interquartile range) or relative frequency (%).

b *P*_trend_ was calculated using age- and sex-adjusted linear regression models.

c Mean (Min − Max).

Table 3 shows the percentage change (and 95 % confidence interval) of different CVD risk makers per 1 point increase in the adolescent lifestyle score. After adjustments of the multivariate adjusted model, inverse associations were observed between adolescence lifestyle score and WC (−4⋅1 % [−5⋅9, −2⋅3 %], *P* = 0⋅0006), WHtR (−4⋅1 % [−5⋅9, −2⋅3 %], *P* = 0⋅0004) and %BF (−9⋅2 % [−13⋅1, −5⋅1 %], *P* = 0⋅0008) in young adulthood. After the additional inclusion of adulthood BMI (conditional model), the associations were no longer significant.

**Table 3.** Associations between adolescence lifestyle score and CVD risk markers in young adulthood^a^

		Basic model		Multivariate adjusted model		Conditional Model	
	*n*	ß (95 % CI)	*P*-value^b^	ß (95 % CI)	*P*-value^b^	ß (95 % CI)	*P*-value^b^
Anthropometric markers							
Waist circumference (cm)	263	−4⋅7 (−6⋅5, −3⋅0)	**<0**⋅**0001**	−4⋅1 (−5⋅9, −2⋅3)	**0**⋅**0001**	0⋅1 (−0⋅8, 1⋅0)	0⋅95
Waist-to-height ratio	263	−4⋅9 (−6⋅6, −3⋅2)	**<0**⋅**0001**	−4⋅1 (−5⋅9, −2⋅3)	**0**⋅**0001**	0 (−0⋅9, 0⋅9)	0⋅98
Body fat content (%)	263	−11⋅3 (−15⋅1, −7⋅3)	**<0**⋅**0001**	−9⋅2 (−13⋅1, −5⋅1)	**0**⋅**0002**	−1⋅3 (−4⋅3, 1⋅8)	0⋅75
Systolic blood pressure (mmHg)	263	−1⋅4 (−2⋅6, −0⋅1)	0⋅15	−1⋅3 (−2⋅6, 0⋅1)	0⋅21	−0⋅9 (−2⋅3, 0⋅5)	0⋅45
Diastolic blood pressure (mmHg)	263	−0⋅8 (−2⋅6, 1⋅0)	0⋅72	−0⋅6 (−2⋅5, 1⋅3)	0⋅83	0⋅2 (−1⋅8, 2⋅2)	0⋅95
Metabolic markers							
Fasting plasma glucose (mg/dl)	228	−0⋅1 (−2⋅4, 2⋅2)	0⋅94	0⋅2 (−2⋅1, 2⋅7)	0⋅95	0⋅6 (−1⋅8, 3⋅2)	0⋅88
Total cholesterol (mg/dl)	228	−2⋅3 (−5⋅6, 1⋅2)	0⋅45	−1⋅7 (−5⋅1, 1⋅8)	0⋅68	−1⋅4 (−5⋅0, 2⋅2)	0⋅76
HDL cholesterol (mg/dl)	228	0⋅4 (−3⋅2, 4⋅2)	0⋅95	0⋅7 (−3⋅1, 4⋅6)	0⋅95	−1⋅1 (−4⋅9, 2⋅8)	0⋅85
LDL cholesterol (mg/dl)	228	−6⋅3 (−11⋅1, −1⋅1)	0⋅08	−5⋅6 (−10⋅6, −0⋅2)	0⋅16	−4⋅6 (−9⋅9, 1⋅0)	0⋅29
Triglycerides (mg/dl)	228	−0⋅4 (−8⋅2, 8⋅0)	0⋅95	−0⋅7 (−8⋅7, 8⋅0)	0⋅94	2⋅6 (−5⋅9, 11⋅7)	0⋅85
Inflammation							
Proinflammatory score^c^	154	−0⋅16 (−0⋅35, 0⋅02)	0⋅24	−0⋅12 (−0⋅31, 0⋅07)	0⋅45	−0⋅02 (−0⋅19, 0⋅16)	0⋅95

Associations were analysed using multiple linear regression. Basic model: adjusted for age and sex; Multivariate adjusted model: Basic model + additionally adjusted for parental education, smoking in the household, exclusive breastfeeding, birth size, maternal overweight and time between measurement of the score and the risk variables; Conditional model: Multivariate adjusted model + additionally adjusted for BMI in adulthood. Values displayed in bold are significant at 0.05 significance level.

a Log-transformed ß values were back transformed.

b Adjusted for multiple testing by Benjamini–Hochberg approach.

c Data are shown as change in tertiles of the proinflammatory score per 1-point increase in the lifestyle score.

Modified lifestyle scores without either diet, MVPA, sedentary behaviour and sleep duration showed significant associations with WC, WHtR and BF (Table 4, *P* < 0⋅05). For the lifestyle score without BMI SDS, no association with WC, WHtR and BF was found (*P* > 0⋅05). For the remaining CVD risk markers (blood pressure, glucose, blood lipids and the proinflammatory score), no association was found for any of the modified lifestyle scores, apart from LDL. The lifestyle score without MVPA is inversely associated with LDL (−8⋅7% [−14⋅6, −2⋅4%], *P* = 0⋅0345).

**Table 4.** Associations between modified version of the adolescence lifestyle score and CVD risk markers in young adulthood^a^

		Adolescent lifestyle score without diet		Adolescent lifestyle score without MVPA		Adolescent lifestyle score without sedentary behaviour		Adolescent lifestyle score without sleep duration		Adolescent lifestyle score without BMI SDS	
	*n*	ß (95 % CI)	*P*-value^b^	ß (95 % CI)	*P*-value^b^	ß (95 % CI)	*P*-value^b^	ß (95 % CI)	*P*-value^b^	ß (95 % CI)	*P*-value^b^
Anthropometric markers											
Waist circumference (cm)	263	−5⋅5 (−7⋅5, −3⋅6)	**<0**⋅**0001**	−5⋅0 (−7⋅1, −2⋅8)	**0**⋅**0001**	−5⋅7 (−7⋅8, −3⋅6)	**<0**⋅**0001**	−4⋅1 (−6⋅0, −2⋅1)	**0**⋅**0004**	0⋅5 (−1⋅5, 2⋅6)	0⋅82
Waist-to-height ratio	263	−5⋅6 (−7⋅5, −3⋅7)	**<0**⋅**0001**	−5⋅0 (−7⋅1, −2⋅8)	**0**⋅**0001**	−5⋅6 (−7⋅6, −3⋅5)	**<0**⋅**0001**	−4⋅0 (−5⋅9, −2⋅0)	**0**⋅**0005**	0⋅2 (−1⋅9, 2⋅2)	0⋅94
Body fat content (%)	263	−11⋅8 (−16⋅0, −7⋅34)	**<0**⋅**0001**	−10⋅1 (−14⋅7, −5⋅1)	**0**⋅**0006**	−10⋅6 (−15⋅2, −5⋅8)	**0**⋅**0002**	−9⋅6 (−13⋅9, −5⋅2)	**0**⋅**0002**	−2⋅1 (−6⋅9, −3⋅0)	0⋅65
Systolic blood pressure (mmHg)	263	−1⋅4 (−2⋅9, 0⋅1)	0⋅21	−1⋅6 (−3⋅1, 0)	0⋅21	−1⋅2 (−2⋅7, 0⋅4)	0⋅37	−1⋅1 (−2⋅5, 0⋅3)	0⋅37	−1⋅1 (−2⋅7, 0⋅5)	0⋅42
Diastolic blood pressure (mmHg)	263	−0⋅8 (−2⋅8, 1⋅3)	0⋅70	−1⋅2 (−3⋅5, 1⋅5)	0⋅57	−0⋅3 (−2⋅5, 1⋅0)	0⋅93	−0⋅6 (−2⋅6, 1⋅4)	0⋅78	−0⋅2 (−2⋅5, 2⋅0)	0⋅94
Metabolic markers											
Fasting plasma glucose (mg/dl)	228	1⋅0 (−1⋅7, 3⋅7)	0⋅70	−0⋅2 (−3⋅1, 2⋅8)	0⋅94	−0⋅4 (−3⋅1, 2⋅4)	0⋅93	−0⋅5 (−3⋅0, 2⋅0)	0⋅88	1⋅4 (−1⋅4, 4⋅4)	0⋅59
Total cholesterol (mg/dl)	228	−2⋅1 (−5⋅8, 1⋅9)	0⋅59	−3⋅7 (−7⋅8, 0⋅5)	0⋅24	−0⋅2 (−4⋅2, 4⋅0)	0⋅95	−0⋅5 (−4⋅1, 3⋅2)	0⋅93	−2⋅8 (−6⋅8, 1⋅4)	0⋅42
HDL cholesterol (mg/dl)	228	1⋅0 (−3⋅2, 5⋅5)	0⋅85	2⋅1 (−2⋅6, 7⋅0)	0⋅64	−0⋅1 (−4⋅5, 4⋅5)	0⋅97	0⋅8 (−3⋅2, 5⋅0)	0⋅89	−0⋅5 (−5⋅0, 4⋅2)	0⋅94
LDL cholesterol (mg/dl)	228	−6⋅6 (−12⋅2, −0⋅7)	0⋅12	−8⋅7 (−14⋅6, −2⋅4)	**0**⋅**0345**	−2⋅8 (−8⋅9, 3⋅7)	0⋅64	−4⋅2 (−9⋅6, 1⋅5)	0⋅37	−6⋅0 (−12⋅1, 0⋅4)	0⋅21
Triglycerides (mg/dl)	228	−4⋅6 (−13⋅0, 4⋅7)	0⋅59	−3⋅2 (−12⋅7, 7⋅2)	0⋅77	−0⋅8 (−10⋅1, 9⋅5)	0⋅95	4⋅2 (−4⋅4, 13⋅6)	0⋅62	0⋅2 (−9⋅4, 10⋅8)	0⋅97
Inflammation											
Proinflammatory score^c^	154	−0⋅18 (−0⋅39, 0⋅02)	0⋅24	−0⋅11 (−0⋅34, 0⋅13)	0⋅63	−0⋅14 (−0⋅33, 0⋅07)	0⋅45	−0⋅13 (−0⋅33, 0⋅07)	0⋅42	−0⋅03 (−0⋅24, 0⋅18)	0⋅93

Associations were analysed using multiple linear regression. MVPA, moderate-to-vigorous physical activity; BMI, body mass index; SDS, standard deviation score. Models were adjusted for age, sex, parental education, smoking in the household, exclusive breastfeeding, birth size, maternal overweight and time between measurement of the score and the risk variables. Values displayed in bold are significant at 0.05 significance level.

a Log-transformed ß values were back transformed.

b Adjusted for multiple testing by Benjamini–Hochberg approach.

c Data are shown as change in tertiles of the proinflammatory score per 1-point increase in the lifestyle score.

### Sensitivity analysis

Our sensitivity analyses with participants (1) who had at least two measurements of all lifestyle factors (*n* 250) and (2) those who provided more correct than potentially underreported reported 3dWR (*n* 244) yielded results comparable to those obtained with the entire sample (*n* 270) (Supplementary Tables S4 and S5).

## Discussion

Our findings indicate that a healthy lifestyle consisting of the five factors favourable diet, physical activity, sedentary behaviour, sleep duration and body weight during adolescence was inversely associated with WC, WHtR and %BF in young adulthood. No association with other CVD risk markers was found, emphasising the role of anthropometrical factors in these age groups. In addition, results of the conditional model indicate that BMI in adulthood plays a major role when analysing CVD risk markers in young adulthood.

To our knowledge, only few studies have investigated the association between combined lifestyle factors in early life and CVD risk markers in later life. In 2012, Laitinen and colleagues have examined to what extent the combination of four health-related behaviours (smoking, BMI, MVPA and diet) and three health factors (cholesterol, blood pressure and glucose) in adolescent participants influence cardiovascular outcomes in adulthood. To quantify ideal cardiovascular health, the authors applied recommendations of the American Heart Association^(61)^. Adherence to the recommendations of the American Heart Association between 12 and 18 years reduced the risk of having hypertension, high LDL cholesterol, metabolic syndrome and high intima-media thickness in adulthood^(39)^. In addition, Meyer and colleagues studied the combined impact of negative lifestyle factors in childhood and concluded that every additional negative lifestyle factor was associated with an increasing risk of an unfavourable cardiovascular risk profile^(40)^. Lifestyle behaviours analysed were obesity, inactivity, little time spent outdoors, skipping breakfast, high media consumption, and parental overweight, inactivity and smoking behaviour. These results together with our findings indicate the considerable relevance of a healthy lifestyle in adolescence for cardiovascular health in adulthood.

The single lifestyle factors considered in our analysis have already been individually linked to anthropometric markers in the literature. For instance, adherence to a healthy dietary pattern has been associated with a lower WC^(62)^ and lower quantity of body fat^(63)^. In addition, regular intense physical activity was associated with both lower %BF^(30,64)^, a more favourable WC^(34)^ and a lower WHtR^(64)^. Furthermore, short sleep duration was inversely associated with WC and %BF^(35,36)^. The associations between obesity markers and individual^(30,34–36,62–64)^ as well as combined lifestyle factors as in our study, underline the importance of diet, activity and sleep in relation to CVD risk markers.

Several studies analysed the relationship between different aspects of a healthy diet^(25–27,49,65–67)^, physical fitness^(28,29,34)^, sleep duration^(35,36)^ or body composition^(37,38,68)^ in adolescence and CVD risk markers in later life. There is robust evidence that a healthy diet in adolescence is related to cardiovascular health later in life^(25–27)^. We decided to include the factors fruits and vegetables^(65)^, whole grain^(49,66)^ sugar-sweetened beverages^(67)^, fish^(69)^, and red meat and sausages^(70)^ in our lifestyle score because these factors seem to play a major role in the maintenance of cardiovascular health. Furthermore, we included MVPA and sedentary behaviour as these measurements were largely used across the literature to determine the relationship between adolescent physical fitness and later CVD risk^(28,29,34)^. In addition, several cross-sectional studies on adolescent physical activity^(71–73)^ or sedentary behaviour^(32,33,71)^ underline the importance of those factors in relation to cardiovascular health. As the fourth lifestyle factor, we included sleep. Recent literature has confirmed that shorter sleep duration is associated with increased risk markers of CVD^(36)^, particularly obesity markers^(35,74)^. We decided to include BMI as part of our lifestyle score, even though BMI might be a consequence of lifestyle rather than a lifestyle factor. To understand the role of each lifestyle factor in adolescence fully, we constructed a modified lifestyle score with only four instead of five lifestyle factors and adjusted the multivariable adjusted model additionally for the omitted lifestyle factor. The results of the regression models showed that a lifestyle score without BMI SDS was not associated with the anthropometric variables WC, WHtR and %BF, but lifestyle scores without either diet, MVPA, sedentary behaviour or sleep duration were associated with WC, WHtR and %BF. Together with our knowledge that associations between the adolescent lifestyle score and WC, WHtR and %BF were no longer significant after adjusting for BMI in adulthood (Table 3, Conditional model), the important role of BMI across the lifespan for later CVD risk was pointed out. Thus, our results indicate that BMI might be an important aspect to consider when investigating the effect on CVD risk.

Scoring systems of combined lifestyle approaches can be based either on the distribution in the underlying population (e.g. median, tertiles) or on references, or even on a combination of both. We did not use population-specific cut-offs because they may limit the comparability with other studies. When reference-based cut-offs are used, they are independent of the underlying population and results from different studies are easily comparable. However, it may happen that the reference-based cut-offs outdate some day and novel references or recommendation levels exist. To the best of our knowledge, we used up-to-date references.

### Strengths and limitations

One of the major strengths of our work is the prospective design of the DONALD study with a follow-up that entails repeated measurements on the same individual. Data on dietary intake was repeatedly measured with 3dWR^(42)^. Due to the self-reported data on dietary intake, underreporting was possible. However, when we excluded underreporters, results of our sensitivity analysis were mainly similar. Furthermore, the questionnaire for MVPA was validated and has been used in a large German survey^(45–48)^. However, measurement error might result due to self-reported data. Nevertheless, any misclassification might be random and the questionnaire can be considered as reliable sources of information. Furthermore, availability of prospectively collected data on several important potential confounders, i.e. early life parameters, anthropometric variables and socio-economic factors of parents strengthened our analyses. Moreover, results from the sensitivity analyses were largely consistent. In addition, false discovery rate was used to account for multiple testing and therefore the possibility of false positive results was limited. Lastly, the score used is transferable to other populations as we have used established national and international cut-offs for all score factors instead of population-based cut-offs.

Nevertheless, there are some limitations. Since there is no uniform definition of lifestyle in the literature, analysing lifestyle patterns might be challenging^(75,76)^. Even though a couple of lifestyle factors were included, additional factors such as daily screen time or social contacts might be of interest. Due to the lack of data on these factors, the inclusion of those variables was not possible. In addition, the questionnaire for sedentary behaviour has not yet been validated, however, used in a nationwide cohort^(46–48)^. Some misclassification with regard to categorisation of individual sedentary behaviour is therefore possible. We were not able to conduct a confirmation analysis in an independent study population and therefore used DONALD participants without CVD risk marker measurements (*n* 265) separately and together with participants with available CVD risk markers (overall *n* 518). Results showed that the lifestyle score was suitable to describe the lifestyle in adolescence. Additionally, we would not expect the developed lifestyle score to misrepresent actual lifestyle, because it is a hypothesis-based score based on national and international recommendations^(16–22)^ instead of an exploratory lifestyle score. Based on the available data, we analysed a convenience sample within the DONALD study. A generalisation of the results to the general population might be limited. In addition, the participants of the DONALD study have a high socio-economic status compared with the general German population^(41)^, which further limits the generalisability of the obtained results. Furthermore, the high socio-economic status might indicate that participants with a very unhealthy lifestyle or high CVD risk, might not be included in the DONALD study. Although our sample is quite homogeneous, which minimises residual confounding, confounding caused by unmeasured covariates, such as family history of CVD, remains possible. These factors may lead to the assumption that the association between the combined adolescent lifestyle score and CVD risk in young adulthood might have been underestimated.

## Conclusion

In conclusion, the results of the present study showed that a combination of lifestyle factors (diet, MVPA, sedentary behaviour and sleep duration, and BMI SDS) in adolescence were inversely associated with WC, WHtR and %BF in German young adults. Special attention should be given to the factor BMI SDS, which seems to play a prominent role in the context of CVD risk markers. For better understanding of the relationship between health-related behaviours and disease occurrence, further studies that include multiple lifestyle factors on the one hand, and investigate their effect throughout the life course, on the other hand, are warranted. A comprehensive understanding would enable early and individually targeted preventive measures for vulnerable individuals.

## Supplementary material

For supplementary material accompanying this paper visit http://dx.doi.org/10.1017/jns.2021.84. click here to view supplementary material

## References

[1] Jiao L, Mitrou PN, Reedy J, (2009) A combined healthy lifestyle score and risk of pancreatic cancer in a large cohort study. Arch Intern Med 169, 764–770. 19398688 10.1001/archinternmed.2009.46 PMC3498842

[2] Leong TI, Weiland TJ, Jelinek GA, (2018) Longitudinal associations of the healthy lifestyle index score with quality of life in people with multiple sclerosis: a prospective cohort study. Front Neurol 9, 874. 30450074 10.3389/fneur.2018.00874 PMC6225868

[3] Lopez-Laguna N, Martinez-Gonzalez MA, Toledo E, (2018) Risk of peripheral artery disease according to a healthy lifestyle score: the PREDIMED study. Atherosclerosis 275, 133–140. 29902701 10.1016/j.atherosclerosis.2018.05.049

[4] Parekh S, Vandelanotte C, King D, (2012) Design and baseline characteristics of the 10 Small Steps Study: a randomised controlled trial of an intervention to promote healthy behaviour using a lifestyle score and personalised feedback. BMC Public Health 12, 179 22405027 10.1186/1471-2458-12-179 PMC3328259

[5] Sotos-Prieto M, Baylin A, Campos H, (2016) Lifestyle cardiovascular risk score, genetic risk score, and myocardial infarction in Hispanic/Latino adults living in Costa Rica. J Am Heart Assoc 5, e004067. 27998913 10.1161/JAHA.116.004067 PMC5210435

[6] Sotos-Prieto M, Bhupathiraju SN, Falcon LM, (2015) A healthy lifestyle score is associated with cardiometabolic and neuroendocrine risk factors among Puerto Rican adults. J Nutr 145, 1531–1540. 25948783 10.3945/jn.114.206391 PMC4478944

[7] Diaz-Gutierrez J, Ruiz-Canela M, Gea A, (2018) Association between a healthy lifestyle score and the risk of cardiovascular disease in the SUN cohort. Rev Esp Cardiol (Engl Ed) 71, 1001–1009. 29287797 10.1016/j.rec.2017.10.038

[8] Bolt HM (2002) Occupational versus environmental and lifestyle exposures of children and adolescents in the European Union. Toxicol Lett 127, 121–126. 12052649 10.1016/s0378-4274(01)00491-x

[9] Cockerham WC (2007) New directions in health lifestyle research. Int J Public Health 52, 327–328. 18368990 10.1007/s00038-007-0227-0

[10] Ford ES, Bergmann MM, Kroger J, (2009) Healthy living is the best revenge: findings from the European Prospective Investigation Into Cancer and Nutrition-Potsdam study. Arch Intern Med 169, 1355–1362. 19667296 10.1001/archinternmed.2009.237

[11] Kessler TA & Alverson EM (2013) Influence of lifestyle, health behavior, and health indices on the health status of underserved adults. J Am Assoc Nurse Pract 25, 674–681. 24170681 10.1002/2327-6924.12027

[12] Barbaresko J, Rienks J & Nothlings U (2018) Lifestyle indices and cardiovascular disease risk: a meta-analysis. Am J Prev Med 55, 555–564. 30241617 10.1016/j.amepre.2018.04.046

[13] Kosti RI, Panagiotakos DB, Mariolis A, (2009) The Diet-Lifestyle Index evaluating the quality of eating and lifestyle behaviours in relation to the prevalence of overweight/obesity in adolescents. Int J Food Sci Nutr 60, 34–47. 19468950 10.1080/09637480802534525

[14] Manios Y, Moschonis G, Papandreou C, (2015) Revised Healthy Lifestyle-Diet Index and associations with obesity and iron deficiency in schoolchildren: the Healthy Growth Study. J Hum Nutr Diet 28, 50–58. 24303926 10.1111/jhn.12183

[15] Papoutsakis C, Papadakou E, Chondronikola M, (2018) An obesity-preventive lifestyle score is negatively associated with pediatric asthma. Eur J Nutr 57, 1605–1613. 28393284 10.1007/s00394-017-1446-7

[16] DGE (2013) The DGE-Nutrition Circle – representation and fundamentals of the food-based recommendations of the German Nutrition Society. Ernaehr Umsch Int 2, 25.

[17] USDA & US HHS (2015) 2015–2020 Dietary Guidelines for Americans, 8th ed. Washington DC: U.S. Department of Health and Human Services and U.S. Department of Agriculture.

[18] WHO (2015) *Sugar Intake for Adults and Children*. Geneva: World Health Organisation

[19] WHO (2010) *Global Recommendations on Physical Activity for Health. Geneva: World Health Organisation*. 26180873

[20] Graf C, Ferrari N, Beneke R, (2017) [Recommendations for physical activity and sedentary behaviour for children and adolescents: methods, database and rationale]. Gesundheitswesen 79, S11–SS9. 28399581 10.1055/s-0042-123701

[21] Paruthi S, Brooks LJ, D'Ambrosio C, (2016) Recommended amount of sleep for pediatric populations: a consensus statement of the American Academy of Sleep Medicine. J Clin Sleep Med 12, 785–786. 27250809 10.5664/jcsm.5866 PMC4877308

[22] Kromeyer-Hauschild K, Wabitsch M, Kunze D, (2001) Perzentile für den body-mass-Index für das kindes-und jugendalter unter heranziehung verschiedener deutscher stichproben. Mschr Kinderheilk 149, 807–818.

[23] Viner RM, Ross D, Hardy R, (2015) Life course epidemiology: recognising the importance of adolescence. J Epidemiol Community Health 69, 719–720. 25646208 10.1136/jech-2014-205300 PMC4515995

[24] Krupp D, Shi L, Egert S, (2015) Prospective relevance of fruit and vegetable consumption and salt intake during adolescence for blood pressure in young adulthood. Eur J Nutr 54, 1269–1279. 25410750 10.1007/s00394-014-0804-y

[25] Berg CM, Lappas G, Strandhagen E, (2008) Food patterns and cardiovascular disease risk factors: the Swedish INTERGENE research program. Am J Clin Nutr 88, 289–297. 18689363 10.1093/ajcn/88.2.289

[26] Bradlee ML, Singer MR, Daniels SR, (2013) Eating patterns and lipid levels in older adolescent girls. Nutr Metab Cardiovasc Dis 23, 196–204. 22417625 10.1016/j.numecd.2011.10.010 PMC3399938

[27] Moore LL, Singer MR, Bradlee ML, (2016) Adolescent dietary intakes predict cardiometabolic risk clustering. Eur J Nutr 55, 461–468. 25724172 10.1007/s00394-015-0863-8 PMC6136431

[28] Hasselstrom H, Hansen SE, Froberg K, (2002) Physical fitness and physical activity during adolescence as predictors of cardiovascular disease risk in young adulthood. Danish Youth and Sports Study. An eight-year follow-up study. Int J Sports Med 23, S27–S31. 12012259 10.1055/s-2002-28458

[29] Ada S G, Olinto MT, Canuto R, (2015) Physical activity in adolescence and abdominal obesity in adulthood: a case-control study among women shift workers. Women Health 55, 419–431. 25893969 10.1080/03630242.2015.1022686

[30] Stamatakis E, Coombs N, Tiling K, (2015) Sedentary time in late childhood and cardiometabolic risk in adolescence. Pediatrics 135, e1432–e1441. 25986017 10.1542/peds.2014-3750 PMC4444802

[31] Hamer M, O'Donovan G, Batty GD, (2020) Estimated cardiorespiratory fitness in childhood and cardiometabolic health in adulthood: 1970 British Cohort Study. Scand J Med Sci Sports 30, 932–938. 32037646 10.1111/sms.13637 PMC7187251

[32] Martinez-Gomez D, Eisenmann JC, Healy GN, (2012) Sedentary behaviors and emerging cardiometabolic biomarkers in adolescents. J Pediatr 160, 104–110, e2. 21839464 10.1016/j.jpeds.2011.06.037

[33] Rey-Lopez JP, Bel-Serrat S, Santaliestra-Pasias A, (2013) Sedentary behaviour and clustered metabolic risk in adolescents: the HELENA study. Nutr Metab Cardiovasc Dis 23, 1017–1024. 22906564 10.1016/j.numecd.2012.06.006

[34] Mielke GI, Brown WJ, Wehrmeister FC, (2019) Associations between self-reported physical activity and screen time with cardiometabolic risk factors in adolescents: findings from the 1993 Pelotas (Brazil) Birth Cohort Study. Prev Med 119, 31–36. 30578907 10.1016/j.ypmed.2018.12.008

[35] Chaput JP, Gray CE, Poitras VJ, (2016) Systematic review of the relationships between sleep duration and health indicators in school-aged children and youth. Appl Physiol Nutr Metab 41, S266–S282. 27306433 10.1139/apnm-2015-0627

[36] Quist JS, Sjodin A, Chaput JP, (2016) Sleep and cardiometabolic risk in children and adolescents. Sleep Med Rev 29, 76–100. 26683701 10.1016/j.smrv.2015.09.001

[37] Tirosh A, Shai I, Afek A, (2011) Adolescent BMI trajectory and risk of diabetes versus coronary disease. N Engl J Med 364, 1315–1325. 21470009 10.1056/NEJMoa1006992 PMC4939259

[38] Lyngdoh T, Viswanathan B, van Wijngaarden E, (2013) Cross-sectional and longitudinal associations between body mass index and cardiometabolic risk factors in adolescents in a country of the African Region. Int J Endocrinol 2013, 801832. 24062771 10.1155/2013/801832 PMC3766579

[39] Laitinen TT, Pahkala K, Magnussen CG, (2012) Ideal cardiovascular health in childhood and cardiometabolic outcomes in adulthood: the Cardiovascular Risk in Young Finns Study. Circulation 125, 1971–1978. 22452832 10.1161/CIRCULATIONAHA.111.073585

[40] Meyer U, Schindler C, Bloesch T, (2014) Combined impact of negative lifestyle factors on cardiovascular risk in children: a randomized prospective study. J Adolesc Health 55, 790–795. 25249036 10.1016/j.jadohealth.2014.07.007

[41] Kroke A, Manz F, Kersting M, (2004) The DGE-nutrition circle – representation and fundamentals of the food-based recommendations of the German Nutrition Society. Current status and future perspectives. Eur J Nutr 43, 45–54. 14991269 10.1007/s00394-004-0445-7

[42] Buyken AE, Alexy U, Kersting M, (2012) [The DONALD cohort. An updated overview on 25 years of research based on the Dortmund Nutritional and Anthropometric Longitudinally Designed study]. Bundesgesundheitsbl 55, 875–884. 10.1007/s00103-012-1503-6 22736170

[43] Schofield WN (1985) Predicting basal metabolic rate, new standards and review of previous work. Hum Nutr Clin Nutr 39, 5–41. 4044297

[44] Goldberg GR, Black AE, Jebb SA, (1991) Critical evaluation of energy intake data using fundamental principles of energy physiology: 1. Derivation of cut-off limits to identify under-recording. Eur J Clin Nutr 45, 569–581. 1810719

[45] Booth ML, Okely AD, Chey TN, (2002) The reliability and validity of the Adolescent Physical Activity Recall Questionnaire. Med Sci Sports Exerc 34, 1986–1995. 12471306 10.1097/00005768-200212000-00019

[46] Lampert T, Sygusch R & Schlack R (2007) [Use of electronic media in adolescence. Results of the German Health Interview and Examination Survey for Children and Adolescents (KiGGS)]. Bundesgesundheitsbl 50, 643–652. 10.1007/s00103-007-0225-7 17514448

[47] Finger JD, Mensink GB, Banzer W, (2014) Physical activity, aerobic fitness and parental socio-economic position among adolescents: the German Health Interview and Examination Survey for Children and Adolescents 2003-2006 (KiGGS). Int J Behav Nutr Phys Act 11, 43. 24656205 10.1186/1479-5868-11-43 PMC3997963

[48] Manz K, Schlack R, Poethko-Muller C, (2014) [Physical activity and electronic media use in children and adolescents: results of the KiGGS study: First follow-up (KiGGS wave 1)]. Bundesgesundheitsbl 57, 840–848. 10.1007/s00103-014-1986-4 24950833

[49] Goletzke J, Buyken AE, Joslowski G, (2014) Increased intake of carbohydrates from sources with a higher glycemic index and lower consumption of whole grains during puberty are prospectively associated with higher IL-6 concentrations in younger adulthood among healthy individuals. J Nutr 144, 1586–1593. 25080538 10.3945/jn.114.193391

[50] Penczynski KJ, Herder C, Krupp D, (2019) Flavonoid intake from fruit and vegetables during adolescence is prospectively associated with a favourable risk factor profile for type 2 diabetes in early adulthood. Eur J Nutr 58, 1159–1172. 29468461 10.1007/s00394-018-1631-3

[51] Diederichs T, Herder C, Rossbach S, (2017) Carbohydrates from sources with a higher glycemic index during adolescence: is evening rather than morning intake relevant for risk markers of type 2 diabetes in young adulthood? Nutrients 9, 591–606. 10.3390/nu9060591 PMC5490570 28604592

[52] Hatziagelaki E, Herder C, Tsiavou A, (2015) Serum chemerin concentrations associate with beta-cell function, but not with insulin resistance in individuals with non-alcoholic fatty liver disease (NAFLD). PLoS ONE 10, e0124935. 25933030 10.1371/journal.pone.0124935 PMC4416815

[53] Herder C, Bongaerts BW, Rathmann W, (2013) Association of subclinical inflammation with polyneuropathy in the older population: KORA F4 study. Diabetes Care 36, 3663–3670. 24009302 10.2337/dc13-0382 PMC3816905

[54] Herder C, Ouwens DM, Carstensen M, (2015) Adiponectin may mediate the association between omentin, circulating lipids and insulin sensitivity: results from the KORA F4 study. Eur J Endocrinol 172, 423–432. 25733068 10.1530/EJE-14-0879

[55] Oluwagbemigun K, Buyken AE, Alexy U, (2019) Developmental trajectories of body mass index from childhood into late adolescence and subsequent late adolescence-young adulthood cardiometabolic risk markers. Cardiovasc Diabetol 18, 9. 30660185 10.1186/s12933-019-0813-5 PMC6339359

[56] Cheng G, Bolzenius K, Joslowski G, (2015) Velocities of weight, height and fat mass gain during potentially critical periods of growth are decisive for adult body composition. Eur J Clin Nutr 69, 262–268. 25005675 10.1038/ejcn.2014.131

[57] Durnin JV & Womersley J (1974) Body fat assessed from total body density and its estimation from skinfold thickness: measurements on 481 men and women aged from 16 to 72 years. Br J Nutr 32, 77–97. 4843734 10.1079/bjn19740060

[58] Calder PC, Ahluwalia N, Albers R, (2013) A consideration of biomarkers to be used for evaluation of inflammation in human nutritional studies. Br J Nutr 109, S1–34. 10.1017/S0007114512005119 23343744

[59] Maldonado G & Greenland S (1993) Simulation study of confounder-selection strategies. Am J Epidemiol 138, 923–936. 8256780 10.1093/oxfordjournals.aje.a116813

[60] Benjamini Y & Hochberg Y (1995) Controlling the false discovery rate: a practical and powerful approach to multiple testing. J R Stat Soc: Ser B (Methodological) 57, 289–300.

[61] Lloyd-Jones DM, Hong Y, Labarthe D, (2010) Defining and setting national goals for cardiovascular health promotion and disease reduction: the American Heart Association's strategic Impact Goal through 2020 and beyond. Circulation 121, 586–613. 20089546 10.1161/CIRCULATIONAHA.109.192703

[62] Cunha CM, Costa PRF, de Oliveira LPM, (2018) Dietary patterns and cardiometabolic risk factors among adolescents: systematic review and meta-analysis. Br J Nutr 119, 859–879. 29644953 10.1017/S0007114518000533

[63] Schneider BC, Dumith SC, Orlandi SP, (2017) Diet and body fat in adolescence and early adulthood: a systematic review of longitudinal studies. Cien Saude Colet 22, 1539–1552. 28538925 10.1590/1413-81232017225.13972015

[64] Riso EM, Kull M, Mooses K, (2018) Physical activity, sedentary time and sleep duration: associations with body composition in 10-12-year-old Estonian schoolchildren. BMC Public Health 18, 496. 29653528 10.1186/s12889-018-5406-9 PMC5899370

[65] Collese TS, Nascimento-Ferreira MV, de Moraes ACF, (2017) Role of fruits and vegetables in adolescent cardiovascular health: a systematic review. Nutr Rev 75, 339–349. 28475799 10.1093/nutrit/nux002

[66] van de Laar RJ, Stehouwer CD, van Bussel BC, (2012) Lower lifetime dietary fiber intake is associated with carotid artery stiffness: the Amsterdam Growth and Health Longitudinal Study. Am J Clin Nutr 96, 14–23. 22623748 10.3945/ajcn.111.024703

[67] Ambrosini GL, Oddy WH, Huang RC, (2013) Prospective associations between sugar-sweetened beverage intakes and cardiometabolic risk factors in adolescents. Am J Clin Nutr 98, 327–334. 23719557 10.3945/ajcn.112.051383 PMC3712546

[68] Owen CG, Whincup PH, Orfei L, (2009) Is body mass index before middle age related to coronary heart disease risk in later life? Evidence from observational studies. Int J Obes (Lond) 33, 866–877. 19506565 10.1038/ijo.2009.102 PMC2726133

[69] O'Sullivan TA, Ambrosini GL, Mori TA, (2011) Omega-3 Index correlates with healthier food consumption in adolescents and with reduced cardiovascular disease risk factors in adolescent boys. Lipids 46, 59–67. 21103948 10.1007/s11745-010-3499-8

[70] Kelishadi R, Pour MH, Zadegan NS, (2004) Dietary fat intake and lipid profiles of Iranian adolescents: Isfahan Healthy Heart Program–Heart Health Promotion from Childhood. Prev Med 39, 760–766. 15351543 10.1016/j.ypmed.2004.02.047

[71] Cristi-Montero C, Chillon P, Labayen I, (2019) Cardiometabolic risk through an integrative classification combining physical activity and sedentary behavior in European adolescents: HELENA study. J Sport Health Sci 8, 55–62. 30719384 10.1016/j.jshs.2018.03.004 PMC6349585

[72] Martinez-Gomez D, Eisenmann JC, Gomez-Martinez S, (2012) Associations of physical activity and fitness with adipocytokines in adolescents: the AFINOS Study. Nutr Metab Cardiovasc Dis 22, 252–259. 21277178 10.1016/j.numecd.2010.07.010

[73] Martinez-Gomez D, Gomez-Martinez S, Ruiz JR, (2012) Objectively-measured and self-reported physical activity and fitness in relation to inflammatory markers in European adolescents: the HELENA Study. Atherosclerosis 221, 260–267. 22265274 10.1016/j.atherosclerosis.2011.12.032

[74] Sun J, Wang M, Yang L, (2020) Sleep duration and cardiovascular risk factors in children and adolescents: a systematic review. Sleep Med Rev 53, 101338. 32619932 10.1016/j.smrv.2020.101338

[75] Jensen M (2007) Defining lifestyle. Environ Sci 4, 63–73.

[76] Jensen M (2009) Lifestyle: suggesting mechanisms and a definition from a cognitive science perspective. Environ Dev Sustain 11, 215–228.

