# Supplementary material for: A lifestyle pattern during adolescence is associated with cardiovascular risk markers in young adults: results from the DONALD cohort study
Source: J Nutr Sci. 2021 Oct 12;10:e92. doi: 10.1017/jns.2021.84 (PMC8532054; doi:10.1017/jns.2021.84)
Supplement: Supplementary file 1 [file S2048679021000847sup001.docx]

**Supplementary material for “A lifestyle pattern during adolescence is associated with cardiovascular risk markers in young adults – results from the DONALD cohort study”**

**S1 Methods:** Additional information on the construction of the lifestyle score

Fruit consumption: Individual portion sizes for fruit consumption in grams/d for each year of age were calculated, whereby fruits were divided into the subcategories whole fruit, dried fruit and fruit juice. Portion sizes can be found in S1 Table. Based on the recommendations of the German Nutrition Society (DGE), one point was assigned if at least 2 portions were consumed by the participants. One portion could be substituted with one portion of juice [1].

Vegetable consumption: Individual portion sizes for fruit consumption in grams/d for each year of age were calculated, whereby vegetables were divided into the subcategories whole vegetables, salad, legumes and vegetable juice. One point was assigned if at least 3 portions of vegetables were consumed by the participants. One portion could be substituted with one portion of juice [1].

Whole grain product consumption: Individual portion sizes for grain consumption in grams/d for each year of age were calculated (S1 Table). A score of one was assigned to participants who consumed ≥1 portion/day, whereas a score of 0 was assigned to participants who consumed <1 portion/day [2]. To classify whole grain products, the definition of the Healthgrain forum was used [3].

Sugar-sweetened beverages: Individual portion sizes for sugar-sweetened beverage consumption in grams/d for each year of age were calculated (S1 Table). Based on the recommendations of the WHO, a score of one was assigned to participants who consumed <1 portion/day, while a score of 0 was assigned to participants who consumed ≥1 portion/day [4].

Fish consumption: Individual portion sizes for fish consumption in grams/d for each year of age were calculated (S1 Table). One point was assigned if at least 1 portion of fish per week was consumed by the participants [1].

Red meat consumption: Individual portion sizes for red meat consumption in grams/d for each year of age were calculated, whereby meat was divided into the subcategories red meat and sausages (S1 Table). A score of one was assigned to participants who consumed ≤6 portions per week, whereas a score of 0 was assigned to participants who consumed >6 portions per week [1].

MVPA: According to a paper from Ridley et al. [38], task-specific metabolic equivalent tasks (MET) values were assigned to each type of physical activity. The duration of activities with a MET value between 3 to 6 (moderate) and a MET value above 6 (vigorous) were summed up and categorized as MVPA. A score of one was assigned for participants having ≥60 minutes/day of MVPA, otherwise a score of 0 was assigned [5].

Sedentary behaviour (range 0-1 point): Participants were categorised according to their daily duration of sedentary behaviour for their age. Based on the recommendations by Graf and colleagues children older than 10 years should have a maximum of 120 minutes of sedentary activities per day. A score of 1 was assigned to participants in line with the recommendation and a score of 0 was assigned to participants being more sedentary than recommended [6].

Sleep duration: A score of 1 was assigned if the sleep duration was within the age-specific references, whereas a score of 0 was assigned if the individual sleep duration was above or below the age-specific reference value [7].

BMI SDS: The body mass index standard deviation score (BMI SDS) was calculated based on the age- and sex-specific LMS method according to a national German reference population [8]. Participants with a BMI SDS outside the threshold for normal weight (≤-2 and >+1), a score of 0 was assigned, whereas a score of 1 was assigned for participants with a BMI SDS within the values for normal weight (-2 to +1) [8].

References

1. DGE, The DGE-Nutrition Circle–representa-tion and fundamentals of the food-based recommendations of the German Nutrition Society. Ernaehrungs Umschau international, 2013. 2(2013): p. 25.

2. USDA, H., 2015–2020 Dietary Guidelines for Americans. 8th Edition. 2015.

3. Ross, A.B., et al., Perspective: A Definition for Whole-Grain Food Products-Recommendations from the Healthgrain Forum. Adv Nutr, 2017. 8(4): p. 525-531.

4. WHO, Sugar intake for adults and children. 2015

5. WHO, Global Recommendations on Physical Activity for Health. 2010.

6. Graf, C., et al., [Recommendations for Physical Activity and Sedentary Behaviour for Children and Adolescents: Methods, Database and Rationale]. Gesundheitswesen, 2017. 79(S 01): p. S11-S19.

7. Paruthi, S., et al., Recommended Amount of Sleep for Pediatric Populations: A Consensus Statement of the American Academy of Sleep Medicine. J Clin Sleep Med, 2016. 12(6): p. 785-6.

8. Kromeyer-Hauschild, K., et al., Perzentile für den Body-mass-Index für das Kindes-und Jugendalter unter Heranziehung verschiedener deutscher Stichproben. Monatsschrift Kinderheilkunde, 2001. 149(8): p. 807-818.

**S2 Methods:** Additional information on the confirmation procedure

We analysed if our developed lifestyle score discriminated individuals according to their actual lifestyle behaviour in a sample of 512 DONALD participants who provided at least one measurement of all lifestyle factors. Fifty-two percent of them (n=270) additionally provided information on CVD risk markers and thus were part of the analysed association between lifestyle score and CVD risk markers. Since the remaining sample size with n=265 participants might be too small for a confirmation analysis of the developed lifestyle score, we tested correctness of the discrimination jointly in a sample of 518 participants (S3 Table) as well as separately for the 265 participants (*P*2_trend_ value in S3 Table). We used multivariable linear regression models adjusted for age and sex to determine the distribution of lifestyle factors within tertiles of the lifestyle score. The results were consistent for both samples and show that the lifestyle score we created was suitable to present participants actual lifestyle in adolescence.

**S1 Table:** Reference values for portion sizes (g/d) by age for different food groups

|  | **8 yrs** | **9 yrs** | **10 yrs** | **11 yrs** | **12 yrs** | **13 yrs** | **14 yrs** | **15 yrs** | **16 yrs** |
| --- | --- | --- | --- | --- | --- | --- | --- | --- | --- |
| **Fruits**  fresh  dried  juice | 95.0  10.0  112.0 | 96.0  10.9  120.0 | 100.0  10.0  126.0 | 102.0  10.0  134.0 | 104.0  10.0  147.6 | 110.0  10.0  156.0 | 118.0  14.7  156.0 | 122.0  11.8  175.0 | 121.0  12.0  173.0 |
| **Vegetables**  fresh  salad legumes juice | 57.6  25.0  57.0  121.8 | 59.0  25.2  55.9  120.0 | 61.8  28.9  51.1  138.0 | 66.3  30.0  63.8  150.0 | 70.5  26.4  65.1  165.0 | 73.6  32.2  61.2  124.0 | 78.3  32.8  70.2  130.0 | 77.0  34.6  73.9  150.0 | 81.1  30.0  77.5  170.0 |
| **Wholegrain products** | 47.0 | 48.0 | 50.0 | 52.3 | 54.0 | 55.0 | 61.0 | 63.0 | 47.0 |
| **Sugar-sweetened beverages** | 200.0 | 200.0 | 200.0 | 208.0 | 225.0 | 250.0 | 250.0 | 280.0 | 200.0 |
| **Fish** | 71.0 | 66.2 | 72.0 | 70.2 | 85.0 | 82.2 | 93.1 | 88.0 | 71.0 |
| **Meat**  Red meat  Sausages | 54.0  20.0 | 59.6  20.0 | 60.0  20.0 | 64.6  21.0 | 70.0  21.8 | 78.5  22.0 | 83.2  21.0 | 80.0  23.0 | 86.1  25.0 |

**S2 Table:** Confirmation analysis of the developed lifestyle score in a sample of 512 participants

|  | Predicted means for different lifestyle factors in tertiles of the adolescent lifestyle score^1^ | | |  |  |
| --- | --- | --- | --- | --- | --- |
|  | Low exposure (T1) | Average exposure (T2) | High exposure (T3) | *P*_trend_^1^ | *P2*_trend_^2^ |
| Fruits (g/d) | 250.5 (224.0; 276.9) | 262.5 (235.3; 289.7) | 339.9 (314.0; 365.9) | **<0.0001** | 0.06 |
| Vegetables (g/d) | 103.5 (93.8; 113.2) | 118.0 (108.1; 128.0) | 133.8 (124.3; 143.3) | **<0.0001** | **0.0001** |
| Wholegrain products (g/d) | 31.0 (25.4; 36.7) | 39.6 (33.8; 45.4) | 59.6 (54.0; 65.2) | **<0.0001** | **<0.0001** |
| Sugar-sweetened beverages (g/d) | 270.9 (233.5; 308.3) | 233.2 (194.6; 271.7) | 146.2 (109.4; 182.9) | **<0.0001** | **<0.0001** |
| Fish (g/d) | 12.4 (9.9; 14.9) | 14.9 (12.3; 17.5) | 15.2 (12.8; 17.7) | 0.12 | 0.35 |
| Red meat (g/d) | 88.1 (82.0; 94.2) | 73.1 (66.8; 79.4) | 67.6 (61.5; 73.6) | **<0.0001** | **0.0001** |
| MVPA (min/d) | 49.8 (45.5; 54.1) | 67.4 (63.0; 71.8) | 76.3 (72.1; 80.5) | **<0.0001** | **<0.0001** |
| Sedentary behaviour (min/d) | 184.4 (177.8; 191.0) | 163.4 (156.7; 170.2) | 124.7 (118.2; 131.2) | **<0.0001** | **<0.0001** |
| Sleep duration (h/d) | 8.8 (8.7; 8.9) | 9.0 (8.9; 9.1) | 9.1 (9.1; 9.2) | **<0.0001** | **0.0003** |
| BMI SDS | 0.5 (0.4; 0.6) | -0.1 (-0.3; 0) | -0.2 (-0.4; -0.1) | **<0.0001** | **<0.0001** |

Values are adjusted least square means (95% confidence interval) of different lifestyle factors in respective tertiles of the lifestyle score. *P*_trend_ values for models are based on multivariable linear regression models adjusted for age and sex. ^1^for n=518 participants with at least one measurement of all lifestyle variables in adolescence (females: 8.5-15.5 years and males: 9.5-16.5 years), including those with outcome measurements (CVD risk marker, n=270). ^2^for n=265 participants with at least one measurement of all lifestyle variables in adolescence but without outcome measurement in young adulthood. MVPA: moderate-to-vigorous physical activity, BMI: body mass index, SDS: standard deviation score.

**S3 Table:** Proportion of participants fulfilling the reference

|  | **T1 (n=92)** | **T2 (n=88)** | **T3 (n=90)** |
| --- | --- | --- | --- |
| Diet (%) | 6.5 | 12.5 | 24.4 |
| MVPA (%) | 3.3 | 17.1 | 25.6 |
| Sedentary behaviour (%) | 1.1 | 4.6 | 16.7 |
| Sleep duration (%) | 38.0 | 58.0 | 62.2 |
| BMI SDS (%) | 38.0 | 78.4 | 91.1 |

MVPA: moderate-to-vigorous physical activity, BMI: body mass index, SDS: standard deviation score.

**S4 Table:** Associations between adolescence lifestyle score and CVD risk markers in young adulthood in participants with at least two measurements of all lifestyle factors (n=250)^1^.

|  |  | Basic model | | Multivariate adjusted model | | Conditional model | |
| --- | --- | --- | --- | --- | --- | --- | --- |
|  | n | ß (95% CI) | *P*-value^2^ | ß (95% CI) | *P*-value^2^ | ß (95% CI) | *P*-value^2^ |
| **Anthropometric markers** |  |  |  |  |  |  |  |
| Waist circumference (cm) | 243 | -5.2 (-7.0 to -3.3) | **<0.0001** | -4.7 (-6.6 to -2.7) | **<0.0001** | 0.1 (-0.9 to 1.0) | 0.92 |
| Waist-to-height ratio | 243 | -5.2 (-7.1 to -3.4) | **<0.0001** | -4.6 (-6.5 to -2.7) | **<0.0001** | 0.1 (-0.9 to 1.0) | 0.92 |
| Body fat content (%) | 243 | -12.4 (-16.4 to -8.3) | **<0.0001** | -10.8 (-14.9 to -6.4) | **<0.0001** | -1.9 (-5.1 to 1.5) | 0.54 |
| systolic blood pressure (mmHg) | 243 | -1.3 (-2.6 to 0) | 0.15 | -1.3 (-2.6 to 0.1) | 0.19 | -0.8 (-2.2 to 0.6) | 0.54 |
| diastolic blood pressure (mmHg) | 243 | -1.0 (-3.0 to 0.9) | 0.54 | -1.0 (-3.0 to 1.0) | 0.54 | 0 (-2.1 to 2.1) | 0.99 |
| **Metabolic markers** |  |  |  |  |  |  |  |
| Fasting plasma glucose (mg/dL) | 210 | -0.4 (-2.8 to 2.1) | 0.92 | 0.2 (-2.3 to 2.8) | 0.92 | 0.6 (-2.1 to 3.3) | 0.91 |
| Total cholesterol (mg/dL) | 210 | -2.1 (-5.6 to 1.5) | 0.54 | -1.5 (-5.1 to 2.3) | 0.73 | -1.1 (-5.0 to 2.9) | 0.82 |
| HDL cholesterol (mg/dL) | 210 | 0.7 (-3.1 to 4.7) | 0.91 | 1.3 (-2.7 to 5.5) | 0.80 | -0.8 (-4.8 to 3.4) | 0.91 |
| LDL cholesterol (mg/dL) | 210 | -5.9 (-11.1 to -0.4) | 0.13 | -5.4 (-10.9 to 0.4) | 0.19 | -4.2 (-10.1 to 2.0) | 0.45 |
| Triglycerides (mg/dL) | 210 | -0.8 (-8.8 to 7.8) | 0.92 | -1.1 (-9.4 to 8.0) | 0.92 | 3.0 (-6.0 to 12.7) | 0.80 |
| **Inflammation** |  |  |  |  |  |  |  |
| Proinflammatory score^3^ | 139 | -0.23 (-0.43 to -0.04) | 0.07 | -0.22 (-0.42 to -0.02) | 0.13 | -0.10 (-0.29 to 0.09) | 0.54 |

^1^Log transformed results were back transformed, ^2^Adjusted for multiple testing by Benjamini-Hochberg approach. ^3^Data are shown as change in tertiles of the proinflammatory score per 1-point increase in the lifestyle score. Associations were analysed using multiple linear regression. Basic model: adjusted for age and sex, Multivariate adjusted model: Basic model + additionally adjusted for parental education, smoking in the household, exclusive breastfeeding, birth size, maternal overweight and time between measurement of the score and the risk variables, Conditional model: Multivariate adjusted model + additionally adjusted for BMI in adulthood.

**S5 Table:** Associations between adolescence lifestyle score and CVD risk markers in young adulthood in participants who provided more correct than potentially underreported 3dWR (n=244)^1^.

|  |  | Basic model | | Multivariate adjusted model | | Conditional model | |
| --- | --- | --- | --- | --- | --- | --- | --- |
|  | n | ß (95% CI) | *P*-value^2^ | ß (95% CI) | *P*-value^2^ | ß (95% CI) | *P*-value^2^ |
| **Anthropometric markers** |  |  |  |  |  |  |  |
| Waist circumference (cm) | 237 | -4.2 (-6.1 to -2.2) | **0.0004** | -3.5 (-5.5 to -1.6) | **0.0035** | -0.1 (-1.0 to 0.9) | 0.97 |
| Waist-to-height ratio | 237 | -4.4 (-6.2 to -2.4) | **0.0004** | -3.6 (-5.5 to -1.6) | **0.0034** | -0.2 (-1.1 to 0.8) | 0.92 |
| Body fat content (%) | 237 | -10.1 (-14.4 to 5.7) | **0.0004** | -8.1 (-12.5 to -3.5) | **0.0044** | -1.3 (-4.6 to 2.1) | 0.85 |
| systolic blood pressure (mmHg) | 237 | -1.4 (-2.8 to 0) | 0.21 | -1.4 (-2.9 to 0) | 0.23 | -1.2 (-2.6 to 0.3) | 0.39 |
| diastolic blood pressure (mmHg) | 237 | -0.7 (-2.7 to 1.4) | 0.85 | -0.5 (-2.6 to 1.6) | 0.87 | 0.2 (-2.0 to 2.3) | 0.97 |
| **Metabolic markers** |  |  |  |  |  |  |  |
| Fasting plasma glucose (mg/dL) | 205 | 0 (-2.5 to 2.6) | 0.97 | 0.4 (-2.2 to 3.1) | 0.93 | 0.7 (-2.0 to 3.4) | 0.87 |
| Total cholesterol (mg/dL) | 205 | 1.8 (-5.4 to 1.9) | 0.73 | -1.1 (-4.8 to 2.7) | 0.85 | -1.2 (-5.0 to 2.2) | 0.85 |
| HDL cholesterol (mg/dL) | 205 | -0.2 (-4.1 to 3.9) | 0.97 | 0.1 (-4.0 to 4.3) | 0.97 | -1.4 (-5.5 to 2.8) | 0.85 |
| LDL cholesterol (mg/dL) | 205 | -5.2 (-10.5 to 0.4) | 0.27 | -4.4 (-9.9 to 1.6) | 0.40 | -3.9 (-9.7 to 2.3) | 0.54 |
| Triglycerides (mg/dL) | 205 | -2.4 (-10.3 to 6.1) | 0.85 | -2.0 (-11.0 to 6.1) | 0.85 | -0.2 (-8.7 to 9.1) | 0.97 |
| **Inflammation** |  |  |  |  |  |  |  |
| Proinflammatory score^3^ | 137 | -0.17 (-0.36 to 0.03) | 0.32 | -0.10 (-0.30 to 0.10) | 0.73 | -0.04 (-0.22 to 0.14) | 0.89 |

^1^Log transformed results were back transformed, ^2^Adjusted for multiple testing by Benjamini-Hochberg approach. ^3^Data are shown as change in tertiles of the proinflammatory score per 1-point increase in the lifestyle score. Associations were analysed using multiple linear regression. Basic model: adjusted for age and sex, Multivariate adjusted model: Basic model + additionally adjusted for parental education, smoking in the household, exclusive breastfeeding, birth size, maternal overweight and time between measurement of the score and the risk variables, Conditional model: Multivariate adjusted model + additionally adjusted for BMI in adulthood.
